# Safety and immunogenicity of shorter interval schedules of the novel oral poliovirus vaccine type 2 in infants: a phase 3, randomised, controlled, non-inferiority study in the Dominican Republic

**DOI:** 10.1016/S1473-3099(23)00519-4

**Published:** 2024-03

**Authors:** Luis Rivera Mejía, Lourdes Peña Méndez, Ananda Sankar Bandyopadhyay, Chris Gast, Sonia Mazara, Katy Rodriguez, Nadia Rosario, Yiting Zhang, Bernardo A Mainou, Jose Jimeno, Gabriela Aguirre, Ricardo Rüttimann

**Affiliations:** a Fundación Dominicana de Perinatologia Pro Bebe, Hospital Universitario Maternidad Nuestra Señora de la Altagracia, Santo Domingo, Dominican Republic; b Clínica Cruz Jiminián, Santo Domingo, Dominican Republic; c Bill & Melinda Gates Foundation, Seattle, WA, USA; d Seattle, WA, USA; e Centers for Disease Control and Prevention, Atlanta, GA, USA; f VaxTrials, Panama City, Panama; g Fighting Infectious Diseases in Emerging Countries, Miami, FL, USA

## Abstract

**Background:**

The novel oral poliovirus vaccine type 2 (nOPV2) is now authorised by a WHO emergency use listing and widely distributed to interrupt outbreaks of circulating vaccine-derived poliovirus type 2. As protection of vulnerable populations, particularly young infants, could be facilitated by shorter intervals between the two recommended doses, we aimed to assess safety and non-inferiority of immunogenicity of nOPV2 in 1-week, 2-week, and 4-week schedules.

**Methods:**

In this phase 3, open-label, randomised trial, healthy, full-term, infants aged 6–8 weeks from a hospital or a clinic in the Dominican Republic were randomly allocated (1:1:1 ratio) using a pre-prepared, computer-generated randomisation schedule to three groups to receive two doses of nOPV2 immunisations with a 1-week interval (group A), 2-week interval (group B), or 4-week interval (group C). The nOPV2 vaccine was given at a 0·1 mL dose and contained at least 10^5^ 50% cell culture infective dose. Neutralising antibodies against poliovirus types 1, 2, and 3 were measured before each immunisation and 4 weeks after the second dose. The primary outcome was the type 2 seroconversion rate 28 days after the second dose, and the non-inferiority margin was defined as a lower bound 95% CI of greater than –10%. Safety and reactogenicity were assessed through diary cards completed by the parent or guardian. The trial is registered with ClinicalTrials.gov, NCT05033561.

**Findings:**

We enrolled 905 infants between Dec 16, 2021, and March 28, 2022. 872 infants were included in the per-protocol analyses: 289 in group A, 293 in group B, and 290 in group C. Type 2 seroconversion rates were 87·5% (95% CI 83·2 to 91·1) in group A (253 of 289 participants), 91·8% (88·1 to 94·7) in group B (269 of 293 participants), and 95·5% (92·5 to 97·6) in group C (277 of 290 participants). Non-inferiority was shown for group B compared with group C (difference in rates –3·7; 95% CI –7·9 to 0·3), but not for group A compared with group C (–8·0; –12·7 to –3·6). 4 weeks after the second nOPV2 dose, type 2 neutralising antibodies increased in all three groups such that over 95% of each group was seroprotected against polio type 2, although final geometric mean titres tended to be highest with longer intervals between doses. Immunisation with nOPV2 was well tolerated with no causal association to vaccination of any severe or serious adverse event; one death from septic shock during the study was unrelated to the vaccine.

**Interpretation:**

Two nOPV2 doses administered 1 week or 2 weeks apart from age 6 weeks to 8 weeks were safe and immunogenic. Immune responses after a 2-week interval were non-inferior to those after the standard 4-week interval, but marked responses after a 1-week interval suggest that schedules with an over 1-week interval can be used to provide flexibility to campaigns to improve coverage and hasten protection during circulating vaccine-derived poliovirus type 2 outbreaks.

**Funding:**

Bill & Melinda Gates Foundation.

## Introduction

Ongoing efforts to eradicate poliomyelitis from the world have resulted in the certified eradication of wild type 2 and 3 poliovirus, with only small pockets of wild type 1 poliovirus transmission endemic to parts of Afghanistan and Pakistan.^1^ Global eradication of wild type 2 poliovirus was certified in September, 2015, but ongoing cases of type 2 poliomyelitis due to circulating vaccine-derived poliovirus (cVDPV2)^2^—334 cases of cVDPV2 compared with 66 cases of wild type 2 in 2021^1^—pose a major risk to the completeness of polio eradication.^3^ The routine use of the trivalent oral poliovirus vaccine (OPV) containing poliovirus types 1, 2, and 3 was replaced with the bivalent OPV containing only types 1 and 3, with an additional one or two doses of inactivated poliovirus vaccines to provide humoral immunity against type 2 poliovirus and to reduce the risk of vaccine-associated paralytic poliomyelitis and vaccine-derived poliovirus from the type 2 component of trivalent OPV. This global switch was implemented in April–May, 2016.^4^ However, continuing and expanding outbreaks of cVDPV2 after the switch means that stockpiles of monovalent OPV type 2 (mOPV2) must be maintained as the only vaccine suitable to respond to these outbreaks since, unlike inactivated poliovirus vaccine, mOPV2 elicits both humoral and intestinal immunity to prevent further transmission of cVDPV2.^5^

**Research in context**

**Evidence before this study**

Since 2011, a global team of researchers and public health professionals, including some authors of this report, has led the process of developing novel oral poliovirus vaccines (nOPVs) in response to increasing numbers of outbreaks from circulating vaccine-derived polioviruses (cVDPVs). The progress has resulted in development of an nOPV vaccine against the predominant strain causing cVDPV; this is the first vaccine to receive emergency use listing by WHO. Development of nOPV type 2 (nOPV2) has been thoroughly documented, including evidence of its safety, tolerability, and immunogenicity in adults, children, and infants, including neonatal infants who received two doses of nOPV2 at 3 days and 4 weeks after birth. Previous studies have evaluated two-dose regimens with a 4-week interval between doses. However, a shorter interval could achieve high prevalence of immunity more rapidly in outbreak settings and could yield improved vaccination coverage in areas with intermittent access due to political or geographical challenges. Furthermore, one report noted a blunting of the immune response to nOPV2 when co-administered with the routine bivalent OPV (types 1 and 3), so early implementation of nOPV2 campaigns preceded or followed by bivalent OPV campaigns will be key for areas with co-circulation of cVDPV types 1 and 2. Because there are no data on administration schedules shorter than 4 weeks, this study aimed to investigate accelerated schedules of 1 and 2 weeks between doses in unvaccinated infants in comparison with the standard 4-week interval.

**Added value of this study**

This is the first study to show that administering two doses of nOPV2 to infants with an interval of only 1 or 2 weeks results in high rates of seroconversion and concentrations of protective neutralising antibodies against type 2 poliovirus. Although the seroconversion rate 4 weeks after the second dose in a 1-week schedule was inferior to the 4-week schedule, the high seroprotection rates and concentrations of neutralising antibodies in both groups suggest that infants would be adequately protected with the 1-week schedule. Furthermore, a 2-week schedule was non-inferior to the 4-week schedule, implying flexibility of the schedule between 2 weeks and 4 weeks.

**Implications of all the available evidence**

Polio is the only disease currently designated as a Public Health Emergency of International Concern, and thus optimisation of early and effective response strategies for polio outbreaks is crucial to minimise the risk of global spread of poliovirus. The overall results of this study add to the increasing volume of data showing the safety and immunogenicity of nOPV2 in infants, with two doses administered 1–4 weeks apart leading to high levels of seroprotective neutralising antibodies. Such schedule flexibility could be particularly important for early interruption of cVDPV2 outbreaks. Unlike inactivated polio vaccines, nOPV2 induces intestinal immunity, which is crucial to reducing transmission of cVDPV2. As nOPV2 is more genetically stable than Sabin-strain monovalent oral polio vaccine type 2 and less likely to revert to neurovirulence, its use lowers the risk of generating new cVDPV2. In the event of a cVDPV2 outbreak, the ability to administer both recommended doses of nOPV2 over a reduced timeframe to vulnerable children could provide logistical and practical advantages for health-care providers responding to outbreaks, particularly in regions with restricted access to vaccines, in areas with co-circulation of other poliovirus types where separate campaigns could be quickly implemented after or before nOPV2 shorter interval campaigns, and in situations with few vaccination opportunities. The data presented here have informed the adoption of flexible schedules for cVDPV2 outbreak response by the WHO Strategic Advisory Group of Experts on Immunization.

Concerns over the risk of seeding further cVDPV2 outbreaks with mOPV2 use led to the development of two novel OPV type 2 vaccine candidates (nOPV2), which were genetically engineered to be more resistant than the Sabin-strain mOPV2 to reversion to neurovirulence.^6^, ^7^ Having been shown to be safe, well tolerated, and immunogenic in extensive testing in adults,^8^, ^9^ children and infants,^10^ and neonates,^11^ the selected nOPV2 candidate became the first vaccine to be authorised for use in outbreak response through the WHO emergency use listing procedure.^12^ Unvaccinated infants—reliant on maternal antibodies for protection—are the most susceptible cohort in outbreak settings, making it essential to generate immunity in this subgroup as rapidly as possible. In settings with logistical constraints, vaccination coverage could be improved with an accelerated administration schedule. Furthermore, when administered simultaneously with bivalent OPV at 6 weeks and 10 weeks of age, blunting of the immune response to nOPV2 has been observed.^13^ The aim of this study was to assess the choice of vaccination intervals shorter than 4 weeks for administration of nOPV2 to infants aged 6–8 weeks at time of first dose, to allow more rapid completion of nOPV2 immunisation campaigns to provide increased flexibility in outbreak response operations, including implementation of additional bivalent OPV campaigns in areas with poliovirus co-circulation.

## Methods

### Study design and participants

This was a phase 3, open-label, randomised, non-inferiority trial done at two sites in the Dominican Republic: Fundación Dominicana de Perinatología Pro Bebe, Hospital Universitario Maternidad Nuestra Señora de la Altagracia, Santo Domingo, and the Clínica Cruz Jiminián, Santo Domingo.

Eligible participants were healthy infants aged 6–8 weeks, born at term with a birthweight of more than 2·5 kg, with no obvious medical conditions likely to affect the immune response (eg, an immunodeficiency disease or condition, receipt of immunotherapies or treatments with corticosteroids, or a family history of immunodeficiency). Main study-specific exclusion criteria included previous receipt of any poliovirus vaccine and presence in the participant's household of any infant younger than 6 months, any children younger than 5 years with incomplete age-appropriate polio vaccination status, or any infant who had received an OPV in the previous 3 months. Other exclusion criteria included congenital defects or serious controlled disease, known allergy to any vaccine component, any evidence of febrile illness on the day of immunisation, or any likelihood in the opinion of the investigator that the participant's parents or guardians would or could not comply with the study protocol. Parents supplied written informed consent to enrol their infant after having the study objectives, risks, and requirements explained to them.

The study protocol was approved by the Independent Ethics Committee, Comité de Bioética de Investigación del Hospital Universitario Maternidad de Nuestra Señora de la Altagracia, Santo Domingo, Dominican Republic, and the national regulatory authority. The study was conducted according to the guidelines of the Declaration of Helsinki and the International Council for Harmonisation of Technical Requirements for Pharmaceuticals for Human Use guideline for Good Clinical Practice.

### Randomisation and masking

After informed consent was obtained and a clinical examination done, infants were enrolled and randomly assigned (1:1:1: ratio) by permuted blocks of varying size to one of three groups to receive two doses of nOPV2 with a 1-week interval (group A), 2-week interval (group B), or 4-week interval (group C). Randomisation was performed using a computer-generated randomisation table prepared previously by Assign Data Management and Biostatistics (Innsbruck, Austria). Due to the nature of the trial, masking was not possible.

### Procedures

The nOPV2 vaccine (batch 2220121) was manufactured by PT Bio Farma (Bandung, Indonesia); nOPV2 was engineered to have improved genetic stability to make the vaccine less prone to reversion to virulence, while retaining the immunogenic properties of the Sabin-strain mOPV2. Each 0·1 mL dose, consisting of two drops administered orally from a supplied dropper, contained at least 10^5^ 50% cell culture infective dose.

**Figure 1:**
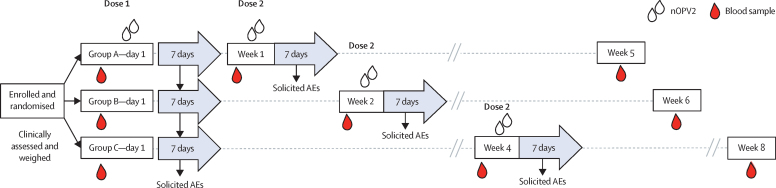
Study design showing schedule of nOPV2 immunisations and blood draws nOPV2=novel oral polio vaccine type 2. AE=adverse event.

At the first study visit, demographic data (age, weight, sex, and race) and any medical history were collected by medical examination and parental interview. Also at the first visit, following a blood draw, all infants received their first immunisation (figure 1). At the next visit a second blood draw preceded a second dose and a third blood sample was obtained at a final visit 4 weeks after the second dose. After each immunisation, infants were monitored for 30 min for any immediate reactions. Parents then used electronic study diaries and supplied thermometers to record any solicited adverse events and axillary temperature for 7 days and any unsolicited adverse events, important medical events, or serious adverse events from the first immunisation to the end of the study, including 6 months of follow-up after dose 2. Telephone contact was made 7 days after each immunisation to review and ensure completion of the study diaries. Solicited (fever, vomiting, atypical crying, drowsiness, loss of appetite, diarrhoea, and irritability) and unsolicited adverse events were graded for severity (mild, moderate, or severe; see appendix pp 2–3 for definitions) with causal association with immunisation determined by the investigator. Serious adverse events and important medical events (see appendix p 3 for definitions) were to be reported immediately to the investigator and to the study sponsor.

All infants received concomitant administrations of their scheduled routine vaccinations according to the recommendations of the Dominican Republic National Immunization Program. These vaccinations included the pentavalent diphtheria, tetanus, whole-cell pertussis, hepatitis B, and *Haemophilus influenzae* type b vaccine and the pneumococcal conjugate vaccine administered at the first study visit, at 6–8 weeks of age, in all groups. However, to avoid interference with study assessments, timings of administration of the rotavirus vaccine and the first dose of the inactivated poliovirus vaccine were altered: the rotavirus vaccine was given at age 8–10 weeks in group A, age 9–11 weeks in group B, and age 11–13 weeks in group C; and the first dose of the inactivated poliovirus vaccine was given at age 11–13 weeks in group A, age 12–14 weeks in group B, and age 14–16 weeks in group C (appendix p 4).

Serum samples prepared immediately after each blood draw were kept below –20°C during storage and shipping to the Polio and Picornavirus Laboratory of the Division of Viral Diseases, Centers for Disease Control and Prevention, Atlanta, GA, USA for measurement of neutralising antibodies against polio types 1, 2, and 3 with the WHO standard microneutralisation assay.^14^, ^15^ Each specimen was run in triplicate, with all specimens from each participant tested in the same assay run to reduce testing bias. Each run contained multiple replicates of a reference antiserum pool and back titrations of the virus to monitor performance variation. Results were expressed as the reciprocal of the log_2_ dilution. A serum sample was considered seropositive if antibodies were present at 8 or more (ie, a log_2_ value of 3); samples with antibody titres of less than 8 were considered seronegative.

### Outcomes

The primary endpoint was the seroconversion rate for polio type 2 neutralising antibodies 28 days after the second dose. The secondary immunogenicity endpoints of the study were the geometric mean titres, geometric mean-fold rises, and seroconversion and seroprotection rates 28 days after the first and second doses for poliovirus types 1, 2, and 3. Secondary safety endpoints were incidence, severity (defined in the appendix; p 4), and causality of serious adverse events, solicited adverse events, unsolicited adverse events, and any important medical events after administration of nOPV2.

### Statistical analysis

The primary objective of the study was to determine whether the seroconversion rate to type 2 poliovirus 4 weeks after immunisation was non-inferior when two doses of nOPV2 vaccine were administered 1 or 2 weeks apart compared with the standard interval of 4 weeks. A participant could seroconvert in one of two ways: for infants who were initially seronegative, seroconversion was defined as becoming seropositive after vaccination; for those who were already seropositive before vaccination, seroconversion was defined as at least a four-fold increase in titre from baseline. Seroconversion was assessed after correcting for waning of maternal antibodies, with an exponential model to estimate the maternally derived antibody titres at post-vaccination timepoints assuming a half-life of 28 days.^16^ The criterion to show non-inferiority was that the lower bound of the two-sided Miettinen-Nurminen 95% CI around each difference (shorter interval minus standard interval) was greater than –10%. To calculate the sample size, we assumed that immune response rates in Dominican Republic infants would be similar to those observed in a previous mOPV2 study done in infants aged 6 weeks in Bangladesh.^16^ Conservatively assuming a cumulative two-dose seroconversion rate of 85% in each group, 270 evaluable participants per group were required to show non-inferiority of each shorter interval to the standard interval, with a one-sided type I error rate of 2·5% and 90% power. Enrolment was increased to 300 per group to account for loss to follow-up or ineligibility for the per-protocol population, the primary population for immunogenicity analyses. Seroconversion rates were also calculated in a subset excluding those whose baseline titres were so high that a four-fold increase would be above the upper limit of quantitation of the assay.

Secondary immunogenicity objectives were assessed through geometric mean neutralising antibody titres, geometric fold-rises, and seroconversion and seroprotection rates 1, 2, or 4 weeks after the first dose and 28 days after the second dose of nOPV2 for poliovirus types 1 and 3. Neutralisation titres were estimated by the Spearman-Kärber method and reported as the reciprocal of the calculated 50% endpoint, with a maximum cutoff of 1448 (log_2_ titre of 10·5), the upper limit of quantitation.

We compared baseline demographic characteristics through Fisher-Freeman Halton and pairwise Fisher's exact tests for categorical variables or Kruskal-Wallis and pairwise Wilcoxon tests for continuous variables. For baseline immunity, a global χ^2^ test for a difference across groups was conducted and, if significant at level 0·10, the model was used to produce pairwise comparisons of each group. The reverse-transformed two-sided 95% CIs for the geometric mean titre ratios, computed assuming asymptotic normality of maximum likelihood parameter estimates, were used to test for a difference between groups that was indicated if the 95% CI excluded a geometric mean titre ratio of 1. Analysis of geometric mean titres was facilitated by a regression model of log_2_ neutralising antibody titres, with left-censoring at assay lower limits of quantitation and right-censoring at assay upper limits of quantitation when necessary.

Safety and reactogenicity data were collated and analysed descriptively for the safety set, which comprised all vaccinated participants. Parameters included the incidence, severity, and causality of solicited and unsolicited adverse events, serious adverse events, and any important medically attended adverse events following administration of nOPV2 in the three different schedules.

Statistical analyses were done under the supervision of the study sponsor by Assign Data Management and Biostatistics (Innsbruck, Austria), with SAS software version 9.3–9.4. The trial is registered with ClinicalTrials.gov (NCT05033561).

### Role of the funding source

ASB is a full-time employee of the funder and was involved in study design, data interpretation, and review of this manuscript. The funder had no other role in the study or manuscript.

## Results

**Figure 2:**
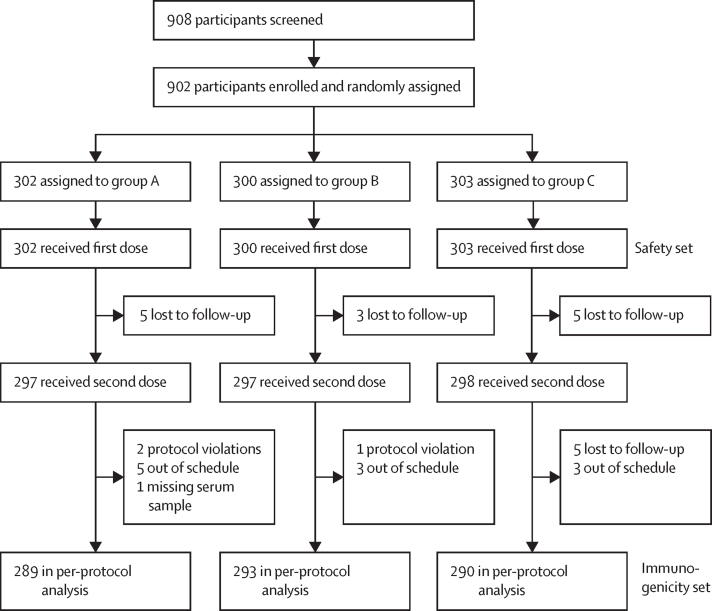
Trial profile showing attrition in the three study groups Group A followed a 1-week vaccination schedule, group B a 2-week schedule, and group C the standard 4-week schedule.

Between Dec 16, 2021, and March 28, 2022, 908 infants were screened. 905 infants were enrolled and randomly allocated to the three study groups (302 in group A, 300 in group B, and 303 in group C). Table 1 shows baseline demographics (sex, mean age, weight, and race) for the three groups. All 905 enrolled infants received their first dose of nOPV2, constituting the safety set. Some infants were reallocated to the different groups for safety analyses according to adherence to the scheduled time-windows; thus participants who received their second dose less than 12 days after the first dose were assigned to group A, participants who received their second dose 12–21 days after the first dose were assigned to group B, and participants who received their second dose more than 21 days after the first dose were assigned to group C. 13 infants were lost to follow-up before receiving their second dose of nOPV2: five from group A, three from group B, and five from group C (figure 2), and were not included in second dose summaries. No participant was withdrawn because of an adverse event.

**Table 1:** Demographics at baseline of the randomised and total vaccinated population

		**Group A (n=302)**	**Group B (n=300)**	**Group C (n=303)**
Sex				
	Male	168 (56%)	162 (54%)	157 (52%)
	Female	134 (44%)	138 (46%)	146 (48%)
Age, days		45·8 (4·1)	45·4 (3·8)	45·8 (4·1)
Weight at visit 1, kg		4·8 (0·6)	4·7 (0·6)	4·7 (0·6)
Race				
	Black or African American	0 (0%)	0 (0%)	1 (0%)
	Hispanic	1 (<1%)	1 (0%)	1 (0%)
	Mixed race	301 (100%)	299 (100%)	301 (99%)

Data are n (%) or mean (SD). Group A had a 1-week interval between vaccinations, Group B a 2-week interval, and Group C a 4-week interval (the current standard interval length).

Seroconversion rates are shown for the three groups overall and according to the baseline serostatus within each group (table 2). The seroconversion rate after the first dose was very low in group A (6·9%; 95% CI 4·3 to 10·5) due to the measurement of the response soon after the immunisation, although 15 (12%) of 122 initially seronegative participants did seroconvert by this timepoint. Seroconversion was observed in 64·3% (95% CI 58·5 to 69·8) of group B participants 2 weeks after the first dose and 78·7% (73·5 to 83·3) of group C participants after 4 weeks. Endpoints for assessing the primary objective were seroconversion rates of 87·5% (95% CI 83·2 to 91·1) in group A, 91·8% (88·1 to 94·7) in group B, and 95·5% (92·5 to 97·6) in group C 4 weeks after the second immunisation (table 2). As the difference between group A (1-week interval) and group C (4-week interval) was –8·0 (–12·7 to –3·6) with the lower bound 95% CI less than –10%, non-inferiority was not shown. The difference between group B (2-week interval) and group C (4-week interval) was –3·7 (–7·9 to 0·3), with the lower bound 95% CI exceeding –10%, showing that immunogenicity after the 2-week interval was non-inferior to the 4-week interval. Seroconversion rates were similar across all three groups among the crucial subgroups of participants who were seronegative at baseline (table 2).

**Table 2:** Type 2 poliovirus seroconversion rates in the overall immunogenicity population and by baseline serostatus

		**Group A**	**Group B**	**Group C**
**Seroconversion rates; overall**				
Before second dose		20/290 (6·9%; 4·3 to 10·5)	189/294 (64·3%; 58·5 to 69·8)	229/291 (78·7%; 73·5 to 83·3)
4 weeks after second dose		253/289 (87·5%; 83·2 to 91·1)	269/293 (91·8%; 88·1 to 94·7)	277/290 (95·5%; 92·5 to 97·6)
Differences in rates from group C		−8·0 (−12·7 to −3·6)	−3·7 (−7·9 to 0·3)	..
Non-inferiority *vs* group C		Non-inferiority not shown	Non-inferiority shown	..
Seroconversion rates in infants with a predicted maternal antibody titre <362*				
Before second dose		20/280 (7·1%; 4·4 to 10·8)	189/283 (66·8%; 61·0 to 72·2)	229/286 (80·1%; 75·0 to 84·5)
4 weeks after second dose		253/282 (89·7%; 85·6 to 93·0)	269/286 (94·1%; 90·7 to 96·5)	277/290 (95·5%; 92·5 to 97·6)
Differences in rates from group C		−5·8 (−10·4 to −1·6)	−1·5 (−5·3 to 2·3)	..
Non-inferiority *vs* group C		Non-inferiority not shown	Non-inferiority shown	..
**Seroconversion according to baseline serostatus**				
Seroconversion before second dose				
	Seronegative	15/122 (12·3%; 7·0 to 19·5)	103/124 (83·1%; 75·3 to 89·2)	111/127 (87·4%; 80·3 to 92·6)
	Seropositive	5/168 (3·0%; 1·0 to 6·8)	86/170 (50·6%; 42·8 to 58·3)	118/164 (72·0%; 64·4 to 78·7)
Seroconversion 4 weeks after second dose				
	Seronegative	115/121 (95·0%; 89·5 to 98·2)	116/124 (93·5%; 87·7 to 97·2)	124/128 (96·9%; 92·2 to 99·1)
	Seropositive	138/168 (82·1%; 75·5 to 87·6)	153/169 (90·5%; 85·1 to 94·5)	153/162 (94·4%; 89·7 to 97·4)

Data are n/N (%; 95% CI) or differences in % (95% CI). We calculated 95% CI for proportions with the Clopper–Pearson exact method. Group A had a 1-week interval between vaccinations, Group B a 2-week interval, and Group C a 4-week interval (the current standard interval length)

* Only a predicted maternal titre at the last blood draw of less than 362 would allow observation of a four-fold increase without being greater than the upper limit of quantitation of 1448.

In some of the initially seropositive infants, it was not possible to show seroconversion of type 2 neutralising antibodies 4 weeks after the second dose as a four-fold higher titre than their predicted titre (based on waning of maternal antibodies) would be higher than the upper limit of quantitation of the assay. This situation affected seven infants in group A and seven in group B but none in group C. When seroconversion rates 4 weeks after the second dose of nOPV2 were calculated without these infants, the rates were 89·7% (95% CI 85·6 to 93·0) in group A, 94·1% (90·7 to 96·5) in group B, and 95·5% (92·5 to 97·6) in group C (table 2). In this subset, the difference between seroconversion rates in groups B and C was –1·5% (–5·3 to 2·3), so meeting the non-inferiority criterion, but the difference between groups A and C was –5·8% (–10·4 to –1·6), with the lower bound 95% CI marginally outside the threshold for non-inferiority.

The high proportions of infants showing seroconversion 4 weeks after the second dose of nOPV2 resulted in over 95% of participants across the three groups having evidence of neutralising antibodies (table 3), which is considered to provide lifelong protection against paralysis due to infection by poliovirus type 2.^17^ This high level of seroprotection was achieved irrespective of whether the participant was seronegative or seropositive before the first dose of nOPV2, with the lowest seroprotection rate occurring in seronegative group B participants given both nOPV2 doses 2 weeks apart (table 3). In comparison, seroprotection rates for poliovirus type 1, which were similar in groups A (58·4%; 95% CI 52·5–64·1), B (54·1%; 48·2–59·9), and C (55·9%; 50·1–61·7) before the first immunisation, had all declined 4 weeks after the second immunisation, to 38·1% (95% CI 32·4–43·9) in group A, 30·7% (25·5–36·3) in group B, and 29·0% (23·8–34·6) in group C (appendix p 5). Similarly, seroprotection rates for type 3 poliovirus, which were lower than for either types 1 or 2 before nOPV2 immunisation in groups A (28·9%; 95% CI 23·7–34·4), B (28·9%; 23·8–34·5), and C (27·8%; 22·8–33·3), also declined 4 weeks after the second dose of nOPV2, to 14·5% (95% CI 10·7–19·1) in group A, 15·0% (11·1–19·6) in group B, and 13·1% (9·4–17·5) in group C (appendix p 5). The seroprotection data for poliovirus type 1 and type 3 substantiate the extent of waning of maternal antibodies against polioviruses over the course of the study. The resulting low concentrations of types 1 and 3 antibodies were not a concern, because infants then received their first dose of inactivated poliovirus vaccine to provide humoral immunity against all three poliovirus types.

**Table 3:** Type 2 poliovirus seroprotection rates* in the overall immunogenicity population and by baseline serostatus

		**Group A**	**Group B**	**Group C**
Seroprotection rate*				
Day 1, before first dose		169/291 (58·1%; 52·2–63·8)	170/294 (57·8%; 52·0– 63·5)	169/295 (57·3%; 50·1–61·7)
Before second dose		150/290 (51·7%; 45·8–57·6)	259/294 (88·1%; 83·8–91·6)	265/291 (91·1%; 87·2–94·1)
4 weeks after second dose		275/289 (95·2%; 92·0–97·3)	282/293 (96·2%; 93·4–98·1)	282/290 (97·2%; 94·6– 98·8)
Seroprotection rate*according to baseline serostatus				
Day 1, before first dose				
	Seronegative	0/122 (0·0%; 0·0–3·0)	0/124 (0·0%; 0·0–2·9)	0/130 (0·0%; 0·0–2·8)
	Seropositive	169/169 (100·0%; 0·0–3·0)	170/170 (100·0%; 97·9–100·0)	165/165 (100·0%; 97·8–100·0)
Before second dose				
	Seronegative	15/122 (12·3%; 7·0–19·5)	103/124 (83·1%; 75·3–89·2)	111/127 (87·4%; 80·3–92·6)
	Seropositive	135/168 (80·4%; 73·5–86·1)	156/170 (91·8%; 86·6–95·4)	154/164 (93·9%; 89·1–97·0)
4 weeks after second dose				
	Seronegative	115/121 (95·0%; 89·5–98·2)	116/124 (93·5%; 87·7–97·2)	124/128 (96·9%; 92·2–99·1)
	Seropositive	160/168 (95·2%; 90·8–97·9)	166/169 (98·2%; 94·9–99·6)	158/162 (97·5%; 93·8–99·3)

Data are n/N (%; 95% CI). We calculated the 95% CI for proportions with the Clopper–Pearson exact method.

* A titre of 8 or higher is considered to be protective against poliovirus infection.

**Figure 3:**
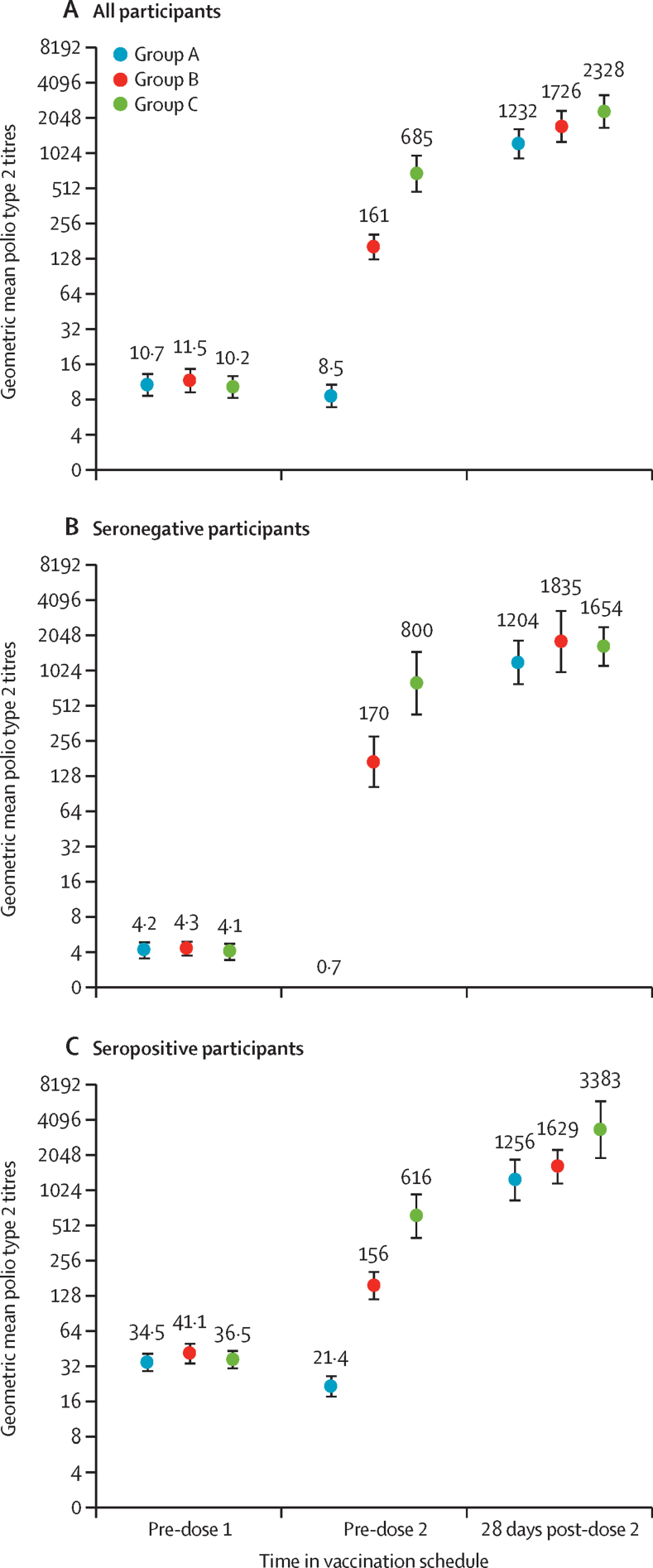
Geometric mean titres of polio type 2 neutralising antibodies Geometric mean titres (with 95% CI bars) in the three study groups (group A=1-week vaccination schedule, group B=2-week schedule, and group C=4-week schedule) before the first and second doses of nOPV2, and 28 days after the second dose for all participants (A), initially seronegative participants (B), and initially seropositive participants (C). Numbers above bars show actual geometric mean titres; n values per group are shown in table 2.

Geometric mean titres in the per-protocol population (figure 3) also show the evolution of the type 2 immune response after the first and second immunisations. From geometric mean titres of 10–11 (log_2_ 3·3–3·5) before the first dose in all three groups, which did not differ significantly (global p value=0·62), in group A there was no increase 1 week after the first dose of nOPV2, in group B after 2 weeks there was an increase to 161 (95% CI 125–209), and in group C after 4 weeks there was an increase to 685 (474–990). There were further increases in all three groups 4 weeks after the second immunisation to final geometric mean titres of 1232 (909–1671) in group A, 1726 (1252–2380) in group B, and 2328 (1654–3275) in group C. Pairwise comparisons indicated a difference only between groups A and C, although this difference was complicated by many observed values exceeding the upper limit of quantitation. Regression modelling assumptions were generally satisfied, although the normality assumption deviated slightly when the proportion of censored observations was high. Estimates of geometric mean titre of responses after dose 2 might therefore be less certain than those for baseline and after dose 1.

When separated according to serostatus, baseline seropositive participants in group A had slightly higher estimated geometric mean titres after doses 1 and 2 than did those who were initially seronegative, whereas the opposite was true in group B. Notably, the geometric mean titre in group C after two doses was about twice as high in initially seropositive participants (3383, 95% CI 1911–5989) than in seronegative participants (1654, 1107–2470). Seroconversion or seroprotection rates did not differ significantly according to sex after two doses, but geometric mean titre estimates were generally higher for males than females 4 weeks after the second dose (appendix p 6).

There was one death during the study: an infant in group C who was hospitalised approximately 3 months after receiving the second dose of nOPV2 died 9 days later from septic shock, an event not considered as causally associated with immunisation. There were 23 infants with a total of 24 non-fatal serious adverse events reported, which consisted of 22 infections and infestations and two congenital disorders including an inguinal hernia (appendix p 8).

**Figure 4:**
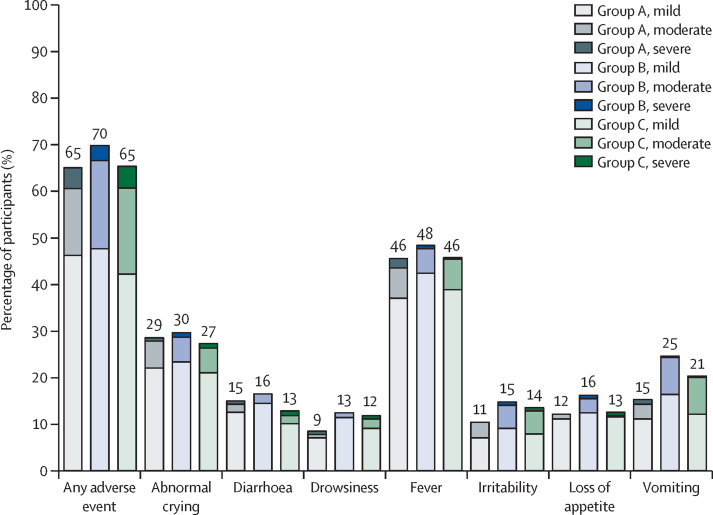
Rates and severity of solicited adverse events after any immunisation in the three study groups Numbers show totals for each group reported for the specified adverse event. Group A had a 1-week vaccination schedule, group B a 2-week schedule, and group C the standard 4-week schedule. Exact numbers for each adverse event (by group and severity) are provided in the appendix (pp 7–8).

Solicited adverse events were reported at rates ranging from 65% to 70% of participants in the three groups (figure 4). These solicited adverse events were mainly mild or moderate in severity and resolved in all cases without sequelae, the most frequent being fever and abnormal crying and temporally associated with concomitantly administered routine vaccinations. Unsolicited adverse events were relatively infrequent, with 135 events reported in 101 participants; these occurred in 26 (9%) of the 298 participants in group A, 35 (12%) of the 304 participants in group B, and 40 (13%) of the 303 participants in group C (appendix p 7). There was one severe unsolicited adverse event in each group, which were already included in the serious adverse event category. Across the three groups there were ten infants each with an unsolicited adverse event considered to be causally related to the immunisation. These mainly consisted of nasal congestion (five cases) and rhinorrhoea (two cases), with individual cases of cough, abdominal pain, and pyrexia.

## Discussion

The primary objective of this study was to determine whether administration of two doses of nOPV2 in shortened schedules with 1 and 2 weeks between doses was non-inferior to the standard 4-week schedule in terms of seroconversion rates achieved 4 weeks after the second dose. Non-inferiority was shown for the 2-week schedule but not the 1-week schedule by a narrow margin. However, all three schedules resulted in high concentrations of neutralising antibodies against type 2 poliovirus, with seroprotection rates exceeding 95%. It was notable that 83% of initially seronegative infants in group B were seropositive 2 weeks after the first dose of nOPV2, showing the rapidity of the protective response as seropositivity is considered to be protective. In the event of a cVDPV2 outbreak, it would appear reasonable to recommend administration of two doses of nOPV2 to infants with at least a 2-week interval between doses, as high rates of immunity were observed in both of the 2-week and 4-week administration schedules.

Before receiving nOPV2, over half the 6–8-week-old infants were seropositive for type 2 poliovirus neutralising antibodies, probably due to maternally derived antibodies, which have an expected half-life of 28 days.^16^ As such, most children would lose immunity by 5 months of age. As observed previously, concomitant administration of nOPV2 with bivalent OPV might result in blunting of the response to nOPV2.^13^ A shorter window with either of the two accelerated immunisation schedules assessed in this study would allow prompt completion of nOPV2 immunisation, enabling flexibility in outbreak response options.

The nOPV2 vaccine was safe and well tolerated in 6–8-week-old infants, confirming previous observations in studies in neonates^12^ and infants,^10^ which themselves showed that nOPV2 was as well tolerated as the Sabin-strain mOPV2 in the standard 4-week schedule. Serious or severe adverse events in this study were rare and no cases were considered to be causally associated with immunisation, consistent with these earlier studies. The increasing proportions of infants reported to have an unsolicited adverse event from group A to group B to group C probably reflects the longer reporting periods in each of the three groups with the longer interval between immunisations.

Our results with nOPV2 are consistent with a previous study in infants in Bangladesh comparing 1-week and 2-week two-dose schedules with the 4-week schedule for mOPV2 administration.^18^ In that study, 161 (93%) of 173 participants in the 1-week group, 169 (96%) of 177 participants in the 2-week group, and 176 (97%) of 181 participants in the 4-week group seroconverted, compared with 253 (88%) of 289 participants in the 1-week group, 269 (92%) of 293 participants in the 2-week group, and 277 (96%) of 290 participants in the 4-week group in our study. Differences in seroconversion rates from the 4-week group in the Bangladesh study were –4·2 (90% CI –7·9 to –0·4) for the 1-week group and –1·8% (90% CI –5·0 to 1·5) for the 2-week group.

With the global withdrawal of type 2 poliovirus from live OPVs used in routine immunisation from April–May, 2016,^4^ and the recommendation to implement immunisation with one or two doses of trivalent inactivated poliovirus vaccine to provide humoral immunity against type 2 poliovirus, infants born after 2016 no longer have vaccine-induced intestinal mucosal immunity against type 2 poliovirus.^5^ We have previously shown induction of intestinal immunity against type 2 poliovirus after one or two doses of nOPV2 in infants from birth^11^ or from 6 weeks of age^19^ when successive vaccine doses were given with the standard 4-week interval. To our knowledge, this study is the first to report safety and immunogenicity of nOPV2 when given at shorter intervals in poliovirus vaccine-naive infants.

Our study has several limitations. We did not assess intestinal immunity and therefore cannot comment on potential effects on reduction of infection, infection duration, or transmissibility upon infection. However, we have no reason to believe that the observed induction of humoral immunity was not accompanied by induction of intestinal immunity in these infants, irrespective of schedule, noting that in previous studies such immunity was measurable after the first dose.^11^, ^19^ Similarly, secondary spread of the vaccine virus that could result in enhancing herd immunity and transmission interruption in the context of polio outbreaks was not assessed in this study. Additionally, vaccine-naive infants were enrolled at age 6–8 weeks for this study, and thus data might not represent the full range of target population age groups (0–5 years) commonly included for polio outbreak response. Another limitation is that we have not assessed waning of induced immunity over time to evaluate differences in duration of immunity induced from different schedules. Furthermore, given the heterogeneity of immune responses to all OPVs documented over decades in different geographies based on host and environmental factors, our results will need to be substantiated with additional data from areas at high risk of polio alongside field use of the vaccine. Finally, selection of the safety set and per-protocol populations for safety and immunogenicity analyses carries risks of bias, but the high retention in this study should have rendered such biases immaterial to the primary conclusions.

nOPV2 is now both stockpiled and is being widely distributed as the primary line of defence against cVDPV2 during outbreaks that continue to occur globally.^1^ This study supports that the vaccine can be used safely to induce immunity in non-immunised infants through an accelerated campaign schedule of less than 4 weeks between doses, the exact choice of which might be dictated by local conditions. Deployment of nOPV2 as a replacement for mOPV2 is a key component of the planning of the Global Polio Eradication Initiative to interrupt cVDPV2 transmission, documented in the updated Polio Eradication Strategy 2022–2026.^20^ The data presented have informed the 2023 recommendation by the WHO Strategic Advisory Group of Experts on Immunization to use a flexible approach with nOPV2 in outbreak responses with intervals from 1 to 4 weeks between doses.^21^ In the event of a cVDPV2 outbreak, the intention is to ensure over 90% of infants at risk have received two vaccinations in two immunisation campaigns within 8 weeks of identification of the circulating poliovirus.^22^ Flexibility to use the shorter schedules rather than the standard 4-week schedule could help to achieve that goal, especially in settings with sporadic access to the population to be immunised (eg, nomadic groups who do not live in areas with established health-care facilities) to rapidly bridge the immunity gap, or in areas of co-circulation, where response against one serotype of polio can be completed faster to mount responses against the other types.

The data from this study, substantiating the safety and tolerability of nOPV2 in 6–8-week-old infants and the high levels of protective immunity induced within 1 or 2 weeks after a first dose and then further increased after a second dose, support the Global Polio Eradication Initiative's cVDPV2 strategies to interrupt cVDPV2 outbreaks. Generation of further data will be helpful to check similar trends in immunogenicity in high-risk settings of polio transmission and to assess the effect of shorter schedules on induction of intestinal immunity by nOPV2, a key factor in preventing further cVDPV2 transmission. The present study adds flexibility to the policy of use of nOPV2 to support its widespread global deployment to more sustainably interrupt cVDPV2 outbreaks.

**This online publication has been corrected. The corrected version first appeared at thelancet.com/infection on January 3, 2024**

## Data sharing

Data for this study will be made available to others in the scientific community upon request. Standard criteria for making data available for valid research projects will be used, following application by suitably qualified researchers. For data access, please contact the Gates Foundation at openaccess@gatesfoundation.org.

## Declaration of interests

We declare no competing interests.
