# Correction to *Lancet Infect Dis* 2023; published online Dec 15. https://doi.org/10.1016/S1473-3099(23)00519-4

**DOI:** 10.1016/S1473-3099(23)00821-6

**Published:** 2024-03

*Rivera Mejía L, Peña Méndez L, Bandyopadhyay AS, et al. Safety and immunogenicity of shorter interval schedules of the novel oral poliovirus vaccine type 2 in infants: a phase 3, randomised, controlled, non-inferiority study in the Dominican Republic.* Lancet Infect Dis *2023; published online Dec 15. https://doi.org/10.1016/S1473-3099(23)00519-4*—In this Article, Chris Gast's affiliation should have been Seattle, WA, USA. This correction has been made to the online version as of Jan 3, 2024, and will be made to the printed version.

