# Supplementary material for: Safety and immunogenicity of shorter interval schedules of the novel oral poliovirus vaccine type 2 in infants: a phase 3, randomised, controlled, non-inferiority study in the Dominican Republic
Source: Lancet Infect Dis. 2024 Mar;24(3):275–84. doi: 10.1016/S1473-3099(23)00519-4 (PMC10881405; doi:10.1016/S1473-3099(23)00519-4)
Supplement: Supplementary appendix [file mmc1.pdf]

THE LANCET
Infectious Diseases

Supplementary appendix

This appendix formed part of the original submission and has been peer reviewed. We post it as supplied by the authors.

Supplement to: Rivera Mejía L, Peña Méndez, L Bandyopadhyay A S, et al. Safety and immunogenicity of shorter interval schedules of the novel oral poliovirus vaccine type 2 in infants: a phase 3, randomised, controlled, non-inferiority study in the Dominican Republic. *Lancet Infect Dis* 2023; published online Dec 15. https://doi.org/10.1016/S1473-3099(23)00519-4.

Supplementary material

Luis Rivera et al - Safety and immunogenicity of different schedules of nOPV2 in infants

Supplementary table 1: Severity definitions for solicited AEs **page 2**

Supplementary table 2: Severity definitions for unsolicited adverse events and SAEs **page 3**

Supplementary table 3: Study immunisation schedule **page 4**

Supplementary table 4: Types 1 and 3 seroprotection rates **page 5**

Supplementary table 5: Type 2 seroconversion, seroprotection and GMTs by gender **page 6**

Supplementary table 6: Solicited AEs with severity (presented in Figure 4) **page 7**

Supplementary table 7: Serious, medically attended, solicited and unsolicited AEs **page 8**

Supplementary Table 1. Severity definitions for solicited adverse events

Adverse Event	Severity	Parameter
Fever*	Mild	37.5°C to 38.0°C
	Moderate	38.1°C to 39.0°C
	Severe	> 39.0°C
Vomiting	Mild	1 episode per 24 hours
	Moderate	2–5 episodes per 24 hours
	Severe	≥ 6 episodes per 24 hours or requiring parenteral hydration
Abnormal crying	Mild	< 1 hour
	Moderate	1–3 hours
	Severe	> 3 hours
Drowsiness	Mild	Sleepier than usual or less interested in surroundings
	Moderate	Not interested in surroundings/did not wake up for a feed/meal
	Severe	Sleeping most of the time or difficult to wake up
Loss of appetite	Mild	Eating less than normal
	Moderate	Missed 1 or 2 feeds/meals completely
	Severe	Refuses ≥ 3 feeds/meals or refuses most feeds/meals
Irritability	Mild	Easily consolable
	Moderate	Requiring increased attention
	Severe	Inconsolable
Diarrhoea	Mild	An increase by 3 bowel movements (above normal or baseline) that are looser than normal per day, with no limitation to activities of daily living
	Moderate	An increase by 4–6 bowel movements (above normal or baseline) that are looser than normal per day, or an increase of 3–6 bowel movements per day (above normal or baseline) that are looser than normal, with some interference with activities of daily living
	Severe	An increase by 7 or more bowel movements per day (above normal or baseline) that are looser than normal per day, or an increase by 3 or more bowel movements per day (above normal or baseline) that are looser than normal, with incapacitating symptoms or interference with activities of daily living, or loose stools with visible red or tarry black blood in stool

* Axillary temperature measured in the evening.

Supplementary Table 2. Severity definitions for unsolicited adverse events

Severity	Parameter
Mild	An adverse event which was easily tolerated by the participant, causing minimal discomfort and did not interfere with everyday activities.
Moderate	An adverse event which was sufficiently discomforting to interfere with normal everyday activities
Severe *	An adverse event which prevented normal, everyday activities (In adults, such an AE would have, for example, prevented attendance at work and necessitating administration of corrective therapy.)

* A severe adverse event was not a serious adverse event (SAE) which was defined as an event that either:

- Resulted in death,
- Was life-threatening, i.e. the participant was at risk of death at the time of the event (e.g. ventricular fibrillation and anaphylaxis). The term does not refer to an event which hypothetically might have caused death if it were more severe,
- Required inpatient hospitalisation or prolongation of existing hospitalisation (Hospitalisation refers to an overnight admission into hospital for the purpose of investigating and/or treating the adverse event. Hospital admissions and/or surgical operations planned before or during the study were not considered adverse events if the illness or disease existed before the participant was enrolled in the study, provided that it did not deteriorate in an unexpected way during the study,
- Resulted in persistent or significant disability/incapacity, i.e., causing substantial disruption of the participant's ability to conduct normal life,
- Was a congenital anomaly/birth defect detected only after being included in the study.

Medically-attended events were those unsolicited adverse events which necessitated a medical visit for treatment of the event.

Important medical events (IME) were medically significant events that did not meet any of the SAE criteria above but required medical or surgical consultation or intervention to prevent the event becoming one of the serious outcomes listed in the SAE definition above.

Supplementary Table 3. Study vaccination schedules compared with routine immunisation schedule in Dominican Republic

	Birth	6–8 weeks	7–9 weeks	8–10 weeks	9–11 weeks	10–12 weeks	11–13 weeks	12–14 weeks	14–16 weeks	24 weeks
**Normal schedule**	BCG HBV	DTPw-HBV-Hib + PCV + Rota + IPV							DTPw-HBV-Hib + PCV + Rota + bOPV	DTPw-HBV-Hib + PCV + IPV
**Group A**	BCG HBV	DTPw-HBV-Hib + PCV **+ nOPV2 (1)**	**nOPV2 (2)**	Rota			IPV		DTPw-HBV-Hib + PCV + Rota + bOPV	DTPw-HBV-Hib + PCV + IPV
**Group B**	BCG HBV	DTPw-HBV-Hib + PCV **+ nOPV2 (1)**		**nOPV2 (2)**	Rota			IPV	DTPw-HBV-Hib + PCV + Rota + bOPV	DTPw-HBV-Hib + PCV + IPV
**Group C**	BCG HBV	DTPw-HBV-Hib + PCV **+ nOPV2 (1)**				**nOPV2 (2)**	Rota*		DTPw-HBV-Hib + PCV + IPV/bOPV	DTPw-HBV-Hib + PCV + IPV

* Second dose of Rota was administered 4 weeks after (15-17 weeks). At 4 months of age all participants completed the immunization schedule according to Dominican Republic EPI.

Supplementary table 4. Types 1 and 3 poliovirus seroprotection rates* in the overall Immunogenicity Per Protocol population

	**Group A (1 week)**		**Group B (2 weeks)**		**Group C (4 weeks)**	
	n / N	**%** [95% CI]	n / N	**%** [95% CI]	n / N	**%** [95% CI]
**Type 1 seroprotection rate ***						
Day 1 – before first dose	170 / 291	**58·4** [52·5, 64·1]	159 / 294	**54·1** [48·2, 59·9]	165 / 295	**55·9** [50·1, 61·7]
Before second dose	146 / 290	**50·3** [44·4, 56·2]	137 / 294	**46·6** [40·8, 52·5]	126 / 291	**43·3** [37·5, 49·2]
4 weeks after second dose	110 / 289	**38·1** [32·4, 43·9]	90 / 293	**30·7** [25·5, 36·3]	84 / 290	**29·0** [23·8, 34·6]
**Type 3 seroprotection rate ***						
Day 1 – before first dose	84 / 291	**28·9** [23·7, 34·4]	85 / 294	**28·9** [23·8, 34·5]	82 / 295	**27·8** [22·8, 33·3]
Before second dose	60 / 290	**20·7** [16·2, 25·8]	60 / 294	**20·4** [16·0, 25·5]	55 / 291	**18·9** [14·6, 23·9]
4 weeks after second dose	42 / 289	**14·5** [10·7, 19·1]	44 / 293	**15·0** [11·1, 19·6]	38 / 290	**13·1** [9·4, 17·5]

* A titre ≥ 8 is considered to be protective against poliovirus infection.
95% CI calculated using Clopper Pearson exact method

Supplementary Table 5. Seroconversion and seroprotection rates and GMTs of poliovirus type 2 neutralising antibodies four weeks after the second dose in the overall Immunogenicity Per Protocol population by gender and GMTs by pre-immunisation serostatus

	Group A (1 week)		Group B (2 weeks)		Group C (4 weeks)	
**Seroconversion rates**	n / N	**%** [95% CI]	n / N	**%** [95% CI]	n / N	**%** [95% CI]
Males	146 / 158	**92·4** [87·1, 96·0]	148 / 156	**94·9** [90·1, 97·8]	139 / 148	**93·9** [88·8, 97·2]
Females	107 / 124	**86·3** [79·0, 91·8]	121 / 130	**93·1** [87·3, 96·8]	138 / 142	**97·2** [92·9, 99·2]
**Seroprotection rates**						
Males	153 / 161	**95·0** [90·4, 97·8]	153 / 159	**96·2** [92·0, 98·6]	142 / 148	**95·9** [91·4, 98·5]
Females	122 / 128	**95·3** [90·1, 98·3]	129 / 134	**96·3** [91·5, 98·8]	140 / 142	**98·6** [95·0, 99·8]
**Geometric mean titres by sex**	**GMT**	**[95% CI]**	**GMT**	**[95% CI]**	**GMT**	**[95% CI]**
Males	1414	[936, 2136]	1852	[1213, 2828]	2841	[1601, 5042]
Females	1035	[661, 1621]	1569	[964, 2554]	1919	[1299, 2835]
**Geometric mean titres by baseline serostatus**						
Seronegative	1204	[771, 1881]	1835	[989, 3405]	1654	[1107, 2470]
Seropositive	1256	[830, 1901]	1629	[1154, 2300]	3383	[1911, 5989

95% CI for proportions calculated using Clopper Pearson exact method

Supplementary Table 6. Solicited adverse events (AE) with severity (presented in Figure 4)

Immunisation interval	Group A<br>1 week	Group B<br>2 weeks	Group C<br>4 weeks
**N =**	298	304	303
**Any solicited AE,** n (%)			
Mild	136 (46)	145 (48)	128 (42)
Moderate	42 (14)	57 (19)	56 (18)
Severe	13 (4)	10 (3)	14 (5)
**Abnormal crying,** n (%)			
Mild	65 (22)	71 (23)	64 (21)
Moderate	17 (6)	16 (5)	16 (5)
Severe	2 (1)	3 (1)	3 (1)
**Diarrhoea,** n (%)			
Mild	37 (13)	44 (14)	31 (10)
Moderate	5 (2)	6 (2)	5 (2)
Severe	2 (1)	0	4 (1)
**Drowsiness,** n (%)			
Mild	21 (7)	35 (12)	28 (9)
Moderate	2 (1)	3 (1)	6 (2)
Severe	2 (1)	0	2 (1)
**Fever,** n (%)			
Mild	109 (37)	129 (42)	118 (39)
Moderate	19 (6)	16 (5)	20 (7)
Severe	6 (2)	2 (1)	1 (0)
**Irritability,** n (%)			
Mild	21 (7)	28 (9)	24 (8)
Moderate	10 (3)	15 (5)	15 (5)
Severe	0	2 (1)	2 (1)
**Loss of appetite,** n (%)			
Mild	33 (11)	38 (13)	35 (12)
Moderate	3 (1)	9 (3)	1 (0)
Severe	0	2 (1)	2 (1)
**Vomiting,** n (%)			
Mild	33 (11)	50 (16)	37 (12)
Moderate	9 (3)	24 (8)	24 (8)
Severe	3 (1)	1 (0)	1 (0)

**Supplementary Table 7.** Serious, medically attended, solicited and unsolicited adverse events			
	**Group A**	**Group B**	**Group C**
**Immunisation interval**	**1 week**	**2 weeks**	**4 weeks**
**N =**	298	304	303
**Serious adverse event,** n (%)			
Death	0	0	1 (0·3)
Hospitalisation	8 (2·7)	7 (2·3)	9 (3·0)
Congenital birth defect	1 (0·3)	1 (0·3)	1 (0·3)
Severe SAE	1 (0·3)	0	0
Moderate SAE	7 (2·3)	7 (2·3)	8 (2·6)
Mild SAE	0	0	0
Related to immunisation	0	0	0
**Specific SAEs,** (n cases)	Pneumonia (1)<br>Bronchiolitis (1)<br>Amoebic dysentery (1)<br>Dengue fever (2)<br>Gastroenteritis (1)<br>Osteomyeltis (1)<br>Inguinal hernia (1)	Pneumonia (2)<br>Bronchiolitis (3)<br>Neck abscess (1)<br>Hereditary spherocytosis (1)	Pneumonia (5)<br>Amoebic dysentery (1)<br>Herpes infection (1)<br>Sepsis (1)<br>Hereditary haemolytic anaemia (1)
**Solicited AEs,** n participants (%) n events			
Any	191 (65·0) 487	212 (69·7) 669	198 (65·3) 585
Severe	13 (4·4) 16	10 (3·3) 10	14 (4·6) 17
**Medically-attended AEs,** n participants (%) n events			
	12 (4·0) 12	11 (3·6) 14	14 (4·6) 18
**Unsolicited AEs,** n participants (%) n events			
Any	26 (8·7) 29	35 (11·5) 51	40 (13·2) 55
Severe	1 (0·3) 1<br>Inguinal hernia	1 (0·3) 1<br>Vomiting	1 (0·3) 1<br>Septic shock
Causally related	3 (1·0) 3<br>Nasal congestion (2)<br>Rhinorrhoea	2 (0·7) 2<br>Nasal congestion<br>Cough	5 (1·7) 5<br>Nasal congestion (2)<br>Rhinorrhoea<br>Abdominal pain<br>Pyrexia
